# Systemic disease in leukocytoclastic vasculitis: a focus on direct immunofluorescence findings^☆^

**DOI:** 10.1016/j.abd.2021.11.009

**Published:** 2022-11-08

**Authors:** Sümeyre Seda Ertekin, Ayşe Esra Koku Aksu, Cem Leblebici, Vefa Aslı Erdemir, Ozan Erdem, Elif Bal Avcı, Mehmet Salih Gürel

**Affiliations:** a Department of Dermatology, Istanbul Training and Research Hospital, University of Health Sciences, Istanbul, Turkey; b Department of Dermatology, Koç University School of Medicine, Istanbul, Turkey; c Department of Pathology, Istanbul Training and Research Hospital, University of Health Sciences, Istanbul, Turkey; d Department of Dermatology, Göztepe Training and Research Hospital, Istanbul Medeniyet University, Istanbul, Turkey; e Department of Dermatology, Başakşehir Çam and Sakura City Hospital, University of Health Sciences, Istanbul, Turkey

**Keywords:** Fluorescent antibody technique, direct, Immunoglobulin M, Immunoglobulin G, Systemic vasculitis, Vasculitis, leukocytoclastic, cutaneous

## Abstract

**Background:**

Direct immunofluorescence (DIF) panels are usually ordered for clinically suspected cutaneous vasculitis, but their positivity rate is variable, and their prognostic significance is not clear to date.

**Objective:**

The study aims to investigate the systemic involvement rate in leukocytoclastic vasculitis (LCV) patients and the potential clinical and laboratory associations with systemic involvement, including DIF findings.

**Methods:**

A retrospective study of patients with histopathologically proven cutaneous LCV examined in the dermatology department between 2013 and 2017 was performed.

**Results:**

Of the 81 patients (mean age, 50.6 years), 42 (52%) were male. The mean time between the appearance of skin lesions and biopsy was 23.1 days, ranging from 2 to 180 days. DIF showed overall positivity of 90.1%, and C3 was the most frequent immunoreactant (82.7%). Any kind of extracutaneous involvement was present in 47 (58%) of patients, with renal involvement being the most frequent (53.1%), followed by articular (18.5%) and gastrointestinal (11.1%) involvement. The presence of renal disease was associated with the detection of IgG in the lesional skin (p = 0.017), and with the absence of IgM in the lesional skin (p = 0.032). There was a significant association between C3 deposition and joint involvement (p = 0.05).

**Study limitations:**

This is a single-center study with a retrospective design.

**Conclusion:**

DIF seems to be a useful ancillary diagnostic tool in the evaluation of cutaneous vasculitis, but the relationship between DIF findings and systemic involvement needs to be further elucidated due to contradictory data in the current literature.

## Introduction

Leukocytoclastic vasculitis is a histopathologic term that defines small-vessel vasculitis in which the perivascular inflammatory infiltrate is composed of neutrophils.^1^ This term is typically used for skin-predominant vasculitis, which most commonly presents with palpable or nonpalpable purpura on the lower extremities. LCV is often idiopathic in nature but may be associated with some underlying conditions, such as infections, exposure to certain medications, connective tissue disorders, or neoplasms.^2^ Leukocytoclastic vasculitis can be limited to the skin, but extracutaneous involvement (renal, gastrointestinal, articular, etc.) is not rare and was reported to be present in 12.5% to 57% of patients with LCV in different studies.^3^, ^4^, ^5^, ^6^, ^7^, ^8^, ^9^

LCV is often mediated by immune complex deposition in the postcapillary venule walls and by activation of the complement system, which results in neutrophil recruitment and finally, destruction of the vessel wall.^10^ Due to the immunological nature of its pathophysiology, LCV has been subject to Direct Immunofluorescence (DIF) studies for many years, and it is recommended to be performed, whenever possible, in the setting of LCV.^11^ DIF can aid in the determination of some specific subtypes of LCV (for example IgA vasculitis) and the identification of the possible underlying etiologies.^6^, ^12^, ^13^ Furthermore, recently some studies tried to determine its possible prognostic significance in LCV, by relating results of DIF examination with specific systemic involvement. The presence of IgM deposition of the vessel walls was associated with renal involvement in some studies,^14^, ^15^ but this finding couldn’t be confirmed by other studies.^6^, ^7^, ^16^, ^17^, ^18^ The prognostic role of DIF studies in LCV is still controversial and currently lacks specificity.^19^

In this study, we aimed to evaluate the potential association between the skin DIF results and extracutaneous involvement in adult LCV patients.

## Methods

### Patient selection

A retrospective review of adult patients (age ≥ 16 years) with histopathologically proven cutaneous leukocytoclastic vasculitis examined in the dermatology department between 2013 and 2017 was performed. Histopathologic diagnosis of LCV was rendered based on the presence of two out of three of the following criteria: (1) Fibrinoid necrosis, (2) Perivascular neutrophilic infiltrate, (3) Disruption and/or destruction of vessel walls by the inflammatory infiltrate.^12^ All of the patients had concomitant DIF analysis, as DIF examination has been routinely performed in the studied clinic for all cases with suspected cutaneous vasculitis since the beginning of 2013. All of the patients had a Complete Blood Test (CBC), serum creatinine level, and urinalysis at the time of their first admission.

### Clinical and laboratory investigations

Medical data abstracted from patients’ records included lesion distribution, the severity of lesions, suspected etiologies, duration of the rash, presence, or absence of any kind of systemic involvement, laboratory findings, and pathology reports (including DIF findings). The severe presentation was defined as presenting vesicles, blisters, ulcers, and/or skin necrosis accompanying palpable/nonpalpable purpuric lesions.

The criteria used for systemic involvement were similar to those used in previous studies.^14^, ^15^, ^16^ Renal involvement was determined through proteinuria, microscopic or macroscopic hematuria, creatinine elevation above baseline, and/or renal involvement confirmed by histological studies. Patients were considered to have Gastrointestinal (GI) tract involvement in the presence of colic-style abdominal pain, hematochezia, and/or positive Fecal Occult Blood Test (FOBT). The existence of arthritis or arthralgia was assessed as joint involvement, only if the onset was recent and if there was no alternative explanation.

Information regarding the following laboratory tests was recorded, whenever they were available: Serological determination for Human Immunodeficiency Virus (HIV), Hepatitis B Virus (HBV), Hepatitis C Virus (HCV); Erythrocyte Sedimentation Rate (ESR); Complement Study (C3, C4); determinations of rheumatoid factor, Antinuclear Antibody (ANA), Antineutrophil Cytoplasmic Antibody (ANCA), cryoglobulins and Fecal Occult Blood Test (FOBT).

### Statistical analyses

Statistical analyses were performed with IBM SPSS Statistics version 24.0 (SPSS Inc., Chicago, IL, USA). Data for qualitative variables were presented as a number (percentage), and data for quantitative variables as mean ± standard deviation or median (minimum-maximum) as appropriate. Pearson’s Chi-Square test was carried out for the comparison of categorical variables; a p-value <0.05 was considered statistically significant.

## Results

### Demographic and clinical characteristics of the patients

From 2013 to 2017, 81 consecutive individuals (39 females, 42 males) were diagnosed as LCV by histopathology in the present study’s hospital, and DIF was performed in all those patients. The demographic and clinical characteristics of the population are summarized in Table 1. The age of the patients ranged from 16 to 90 years with a mean age of 50.6 years. The mean duration of rash from the reported onset to the time of biopsy was 23.1 days (range 2‒180 days). The commonest site was the lower extremity which was involved in all patients (n = 81, 100%). Thirty-seven (45.7%) of the cases had lesions above the waist, which were distributed on the upper extremities (n = 32, 39.5%), trunk (n = 22, 27.2%) and head and neck (n = 2, 2.5%). While most of the patients presented with petechiae, purpura, or macules, 35 (43.2%) patients showed severe skin involvement in the form of bullae, ulcers, and/or necrosis.

**Table 1:** Demographic and clinical findings of the patients.

Characteristics	Patients (n = 81) n (%)
**Age, mean ± SD; median (min.‒max.), years**	50.6 ± 20.3; 52 (16–90)
**Sex**	
Female	39 (48.1)
Male	42 (51.8)
**Duration of rash to biopsy, mean ± SD; median (min.‒max.), days**	23.1 ± 34.4; 10 (2–180)
**Site of involvement**^a^	
Head & neck	2 (2.5)
Trunk	22 (27.2)
Upper extremities	32 (39.5)
Arm	29 (35.8)
Hand	22 (27.2)
Lower extremities	81 (100)
Thigh	40 (49.4)
Leg	81 (100)
Foot	76 (93.8)
**Skin lesions above waist**	37 (45.7)
**Severe cutaneous involvement (bullae, ulcer, necrosis)**	35 (43.2)
**Suspected etiologies**	
Idiopathic	48 (59.3)
Concomitant infection + antibiotic use	13 (16)
Infection	9 (11.1)
Neoplasms	6 (7.4)
Drug exposure	5 (6.2)
**Systemic involvement, any**	47 (58)
Renal	43 (53.1)
Joint	15 (18.5)
Gastrointestinal	9 (11.1)
Peripheric nerve	1 (1.2)

a Total percentage may exceed 100% because of multiple involvement.

The most common underlying cause was concomitant infection and systemic antibiotic use (16%), followed by infection (11.1%), neoplasms (7.4%), and drug exposure (6.2%). No etiological association or predisposing factors could be identified in 48 (56.3%) patients which were classified as idiopathic LCV.

Any kind of extracutaneous involvement was present in 47 (58.0%) of patients. Articular involvement was noted in 15 patients (18.5%), renal involvement in 43 (53.1%), and gastrointestinal involvement was noted in 9 (11.1%). Only one patient (1.2%) had neurologic involvement in the form of peripheral neuropathy.

### Laboratory and immunofluorescence findings

The most common laboratory alteration was elevated ESR, which was observed in 45 out of 64 patients (70.3%). Leukocytosis (WBC > 11.0 × 10^9/L) was detected only in 24.7% of patients. All laboratory and immunofluorescence findings were summarized in Table 2. Renal involvement was detected as proteinuria in 31 of 81 patients (38.3%) and microscopic or macroscopic hematuria in 42 patients (51.9%). In 30 cases (37%), proteinuria and hematuria were detected together. Only 11 patients (13.6%) had serum creatinine elevation above baseline. Patients with renal involvement were referred to nephrology for follow-up. GI tract involvement was detected as FOBT positivity in 6 of 57 patients (10.5%).

**Table 2:** Laboratory and direct immunofluorescence of skin lesion characteristics of the patients.

Characteristics	Patients n/total (%)
**High ESR**	45/64 (70.3)
**WBC alteration (leukocytosis)**	20/81 (24.7)
**Renal dysfunction**	43/81 (53.1)
Proteinuria	31/81 (38.3)
Hematuria	42/81 (51.9)
Hematuria + proteinuria	30/81 (37)
Serum creatinine elevation above baseline	11/81 (13.6)
**Positive fecal occult blood test**	6/57 (10.5)
**Positive ANA**	4/50 (8)
**Low C3/C4**	4/45 (8.9)
**Positive rheumatoid factor**	3/35 (8.6)
**Positive ANCA**	1/41 (2.4)
**Cryoglobulinemia**	2/32 (6.3)
**HIV (+)**	1/44 (2.3)
**Hepatitis B (+)**	1/46 (2.2)
**Hepatitis C (+)**	0/46 (0)
**Positive immunoreactant, any**	73/81 (90.1)
IgA	35/81 (43.2)
IgG	18/81 (22.2)
IgM	19/81 (23.5)
C3	67/81 (82.7)
Fibrinogen	59/81 (72.8)

ESR, Erythrocyte Sedimentation Rate; WBC, White Blood Cells; C, Complement, ANA, Anti-Nuclear Antibody; ANCA, Antineutrophil Cytoplasmic Antibodies; HIV, Human Immunodeficiency Virus; Ig, Immunoglobulin.

DIF was positive in 73 patients (90.1%), with C3 being the most common immunoreaction deposited in the wall of blood vessels (82.7%), followed by fibrinogen (72.8%), IgA (43.2%), IgM (23.5%) and IgG (22.2%). Table 3 shows the relation of DIF positivity with the duration of rash from the reported onset to the time of biopsy. Although statistically not significant; the DIF positivity rate was higher in biopsies performed within 7 days (95.8%), compared to biopsies performed between 7‒28 days (89.5%) and later than 28 days (84.2%) (p = 0.440).

**Table 3:** Relation of time of biopsy and direct immunofluorescence positivity.

Timing of biopsy	DIF positivity		p-value
	Yes, n (%)	No, n (%)	
< 7 days	23 (95.8%)	1 (4.2%)	0.440
7–28 days	34 (89.5%)	4 (10.5%)	
> 28 days	16 (84.2%)	3 (15.8%)	

### Association between some clinical, laboratory and immunofluorescence parameters and systemic involvement

The presence of lesions above the waist (upper extremities, trunk, face) was found to be associated with renal (p = 0.017) and GI involvement (p = 0.04), but not with joint involvement. Severe cutaneous involvement in the form of bullae, ulcers, and/or necrosis was not found to be a significant predictor of the renal, gastrointestinal tract, or joint involvement disease. We found no relationship between the presence of an etiological factor (infection, drug exposure, and/or malignancy) and any kind of systemic involvement (Table 4).

**Table 4:** Association between clinical, laboratory and immunofluorescence parameters and systemic involvement.

Characteristics	Renal involvement		p-value	Joint involvement		p-value	GIS involvement		p-value
	No (n = 38)	Yes (n = 43)		No (n = 66)	Yes (n = 15)		No (n = 72)	Yes (n = 9)	
**Lesions above waist**	12 (31.6)	25 (58.1)	**0.017**	28 (42.4)	9 (60)	0.217	30 (41.7)	7 (77.8)	**0.040**
**Severe cutaneous involvement (bullae, ulcer, necrosis)**	13 (34.2)	22 (51.2)	0.124	29 (43.9)	6 (40)	0.781	30 (41.7)	5 (55.6)	0.428
**Presence of etiologic factor**	17 (44.7)	17 (39.5)	0.638	26 (39.4)	8 (53.3)	0.323	30 (41.7)	4 (44.4)	0.874
**ESR alteration (n = 64)**	19/31 (61.3)	25/33 (75.8)	0.212	33/52 (63.5)	11/12 (91.7)	0.057	38/56 (67.9)	6/8 (75)	0.683
**WBC alteration (leukocytosis)**	6 (15.8)	14 (32.6)	0.081	15 (22.7)	5 (33.3)	0.390	17 (23.6)	3 (33.3)	0.524
**Positive immunoreactant, any**^a^	35 (92.1)	38 (88.4)	0.574	58 (87.9)	15 (100)	0.340	67 (93.1)	6 (66.7)	**0.041**
IgA	16 (42.1)	19 (44.2)	0.850	29 (43.9)	6 (40)	0.781	33 (45.8)	2 (22.2)	0.178
IgG	4 (10.5)	14 (32.6)	**0.017**	12 (18.2)	6 (40)	0.067	17 (23.6)	1 (11.1)	0.395
IgM	13 (34.2)	6 (14)	**0.032**	16 (24.2)	3 (20)	0.726	18 (25)	1 (11.1)	0.354
C3	32 (84.2)	35 (81.4)	0.738	52 (78.8)	15 (100)	**0.050**	61 (84.7)	6 (66.7)	0.182
Fibrinogen	30 (78.9)	29 (67.4)	0.245	47 (71.2)	12 (80)	0.490	53 (73.6)	6 (66.7)	0.659

GIS, Gastrointestinal System; ESR, Erythrocyte Sedimentation Rate; WBC, White Blood Cells; C, Complement; Ig, Immunoglobulin.

a Total percentage may exceed 100% because of multiple positivity.

There was no link between ESR alteration or leukocytosis and any kind of systemic involvement.

The presence of renal disease was associated with the detection of IgG in the lesional skin (p = 0.017), whereas the absence of IgM in the lesional skin (p = 0.032). There was a significant association between C3 deposition and joint involvement (p = 0.05). Apart from these, there was no association between the existence or absence of any immunoreaction and systemic involvement (Table 4).

## Discussion

The evaluation of the patient with cutaneous lesions suspicious of vasculitis should include a detailed anamnesis and physical examinations with some specific laboratory tests not to miss a potential systemic involvement. Treatment approach and follow-up schedules vary depending on whether there is systemic involvement or not. In the present study, we have retrospectively searched for the systemic involvement rate in LCV patients and potential clinical and laboratory associations with systemic involvement, including DIF findings.

Systemic involvement was documented in 58% of all LCVs in the present study, which is in the upper range of previous reports: from 12.5% to 57% depending on the design of studies and the inclusion criteria of the patients.^4^, ^6^, ^7^, ^8^, ^9^ Joint involvement is usually reported to be the most frequent site of involvement, followed by renal and GI involvement.^8^, ^9^ However, in this study, renal involvement was the most frequent systemic involvement. The relatively lower renal involvement rates in some other studies could be explained by the strict criteria they used for defining renal involvement, such as the necessity of histological-proven nephropathy.^6^

In most centers, DIF analysis is routinely performed in clinically suspected cases of cutaneous vasculitis. Small vessel vasculitis of the skin is mediated by immune complex deposition in the post-capillary venules. Circulating antigens due to drug exposure, infectious agents, connective tissue disease, or neoplasia are bound by antibodies, forming immune complexes, which then can be detected by direct immunofluorescence studies.^20^ The overall positivity rate of DIF in LCV cases varies in the literature from 39% to as high as 97%.^17^, ^21^, ^22^, ^23^, ^24^ In the present study, we found positive DIF for at least one immunoglobulins and/or complement and/or fibrinogen in 90.1% of the cases, compatible with the previous reports. The most common positive immunoreactant was C3, and the most common positive immunoglobulin was IgA. Regarding the positivity pattern, the present findings are in line with the current literature where C3 is the most common immunoreaction in almost all of the studies.^6^, ^17^, ^22^, ^25^^,^^26^ Although the exact reason for this finding is not known, we can assume that it may be related to the timing of the skin biopsy as immunoglobulins tend to be disappearing faster compared to the complement, and C3 is known to be deposited in relatively later lesions of vasculitis.^9^, ^27^

For an ideal evolution of DIF specimens, it is advised to choose a newer lesion, between 8 h and 24 h of age, to biopsy. It has been shown that the DIF positivity rates drop concerningly in older lesions, especially if they are >1 week old. In a study reported by Nandeesh et al., the DIF positivity rate was 85% if the biopsy was taken within one week of onset of symptoms, whereas it was only 14% if was taken after a week.^22^ In the present study DIF positivity rate was found to be higher if the samples were taken early during the course of the disease (95.8% for <7 days vs. 84.2% for >28 days), however, the difference was not as obvious as it is in the report of Nandeesh et al. But these results should be interpreted carefully, because in the report of Nandeesh et al., as in ours, the exact age of each individual lesion that was biopsied could not be detected, due to retrospective design of both studies. Instead, the time from the appearance of the first lesion to the biopsy was included in the analyses. In the present clinic, we always tend to find a fresh appearing individual skin lesion to biopsy, even in the setting of a longer disease course. We believe that the results of the present study are interesting, as it showed that even if the vasculitic skin rash is present for a longer period of time, DIF can still be positive and diagnostic, especially if we manage to find a fresh appearing lesion to biopsy.

The most frequent etiological causes of LCV are infections, drug exposure, neoplasm, and connective tissue disorders, while almost half of the cases are idiopathic in which no etiological cause can be determined.^28^, ^29^, ^30^ Consistent with the previous reports, in the present study, idiopathic cases ranked at the top, at 59.3%, and concomitant infections and antibiotic use were second, at 16%.

We have investigated if some clinical features could be a clue for systemic involvement in patients with LCV. In some previous reports, presenting cutaneous vasculitic lesions above the waist has been found to point out renal or gastrointestinal involvement in HSP patients.^20^, ^27^, ^31^ Severe cutaneous involvement with vesiculobullous lesions was found to be indicative of systemic involvement in some reports, but this correlation could not be validated in some others.^14^, ^28^, ^32^ In the present study, the presence of lesions above the waist was significantly associated with both renal and GI involvement, thus emphasizing the importance of a thorough clinical exam. However, severe cutaneous involvement (in form of bullae, ulcers, and/or necrosis) was not associated with systemic involvement. In other words, in the present study systemic involvement was not related to the severity or type of skin lesions, but to their localization.

For patients diagnosed with LCV, any laboratory changes possibly indicating systemic involvement are important. In the literature, high ESR was reported to be the most frequently seen laboratory finding among LCV patients.^4^, ^26^, ^28^ In some of those studies, high ESR was associated with systemic involvement, whereas in some others no such relationship could be found. Similarly, high ESR was the most common laboratory alteration in the present study, being present in 70.3% of patients, and neither it nor leukocytosis was related to systemic involvement.

Numerous studies have attempted to correlate DIF findings with systemic disease in patients with cutaneous vasculitis. The findings of the previous studies related to DIF findings in LCV are summarized in Table 5.^6^, ^7^, ^9^, ^17^^,^^23^, ^26^ The presence of IgA in the lesional skin of LCV patients was associated with renal involvement in two different case series.^7^, ^17^ However, Takatu et al. couldn’t revalidate this finding, but instead, they found that C3 deposition at the blood vessel wall was related to renal involvement.^6^ In some other reports, no association was found between any extracutaneous involvement and DIF results.^9^, ^26^ In the present study, the presence of IgG in lesional skin was associated with renal involvement and the presence of C3 with joint involvement. Furthermore, the absence of IgM was associated with renal involvement. Unlike ours, in some studies which only included HSP patients, the authors concluded that there was a positive correlation between IgM deposition and renal involvement.^14^, ^15^

**Table 5:** Findings of previous studies about the relationship between DIF findings and systemic involvement in LCV patients.

	Number of included patients	Systemic involvement rate (any type)	Renal involvement rate	Gastrointestinal involvement rate	Joint involvement rate	DIF positivity rate	Most common positive immunoreactant in DIF examination	Correlation of DIF findings with systemic involvement
Sais et al.^26^	160	20%* (*Other than the joint involvement)	N/A	N/A	N/A	84.3%	C3, followed by IgA	No correlation was found between DIF and systemic involvement
Barnadas et al.^17^	50	N/A	50%	N/A	N/A	92%	C3, followed by IgA	IgA deposition was related to renal involvement
Alalwani et al.^7^	88	50.9%	21.7%	15.1%	34.9%	70.5%	IgA was the most common Immunoglobulin (no info about C3)	IgA deposition was related to renal and gastrointestinal involvement.
Takatu et al.^6^	282	12.5%	N/A	N/A	N/A	70,2%	C3, followed by IgA	C3 deposition was related to hematuria and renal involvement
Gülseren et al.^9^	68	N/A	52%	28%	57%	53%	C3, followed by IgA	No association was found between DIF results and extracutaneous involvement
Lath et al.^23^	198	29.4%	N/A	10%	18.8%	60%	IgA, followed by C3	N/A
Present study, 2021	81	58%	53.1%	11.1%	18.5%	90.1%	C3, followed by IgA	IgG deposition was associated with renal involvement.
								Absence of IgM deposition was associated with renal involvement.
								C3 deposition was associated with joint involvement.

N/A, Not Available: It is used when related information was not found in the study.

Limitations of this study include a single-center study with a relatively small sample size, though comparable to other DIF-related studies in LCV. Due to the retrospective design of the study not all patients had exactly the same work-up performed, which may lead to an overestimation of idiopathic cases in the present series. Another important limitation is the lack of long-term follow-up information on patients with systemic involvement, so their prognosis remains unknown.

## Conclusion

In conclusion, DIF seems to be a useful ancillary diagnostic tool in the evaluation of cutaneous vasculitis, being positive in more than 90% of all patients. Systemic involvement was not rare and was detected in 58% of patients. Deposition of IgG and absence of IgM were associated with renal involvement, whereas deposition of C3 was associated with joint involvement. In light of the results of this study and contradictory data in the literature, we believe that the potential relationship between DIF findings and systemic involvement needs to be further elucidated, as differences in the results may generate discordant interpretations. Distribution of the skin lesions above the waist was a reliable indicator of renal and gastrointestinal disease in adults with LCV, thus emphasizing the importance of a thorough clinical exam.

## IRB approval status

The research protocol received approval from the Research Ethics Committee of the Istanbul Training and Research Hospital (IRB number: IEAH-KAEK-2830).

## Financial support

None declared.

## Authors' contributions

Sümeyre Seda Ertekin: Study concept and design; data collection, or analysis and interpretation of data; statistical analysis; writing of the manuscript or critical review of important intellectual content; effective participation in the research guidance; intellectual participation in the propaedeutic and/or therapeutic conduct of the studied cases; critical review of the literature; final approval of the final version of the manuscript.

Ayşe Esra Koku Aksu: Study concept and design; writing of the manuscript or critical review of important intellectual content; effective participation in the research guidance; intellectual participation in the propaedeutic and/or therapeutic conduct of the studied cases; final approval of the final version of the manuscript.

Cem Leblebici: Study concept and design; data collection, or analysis and interpretation of data, effective participation in the research guidance; final approval of the final version of the manuscript.

Vefa Aslı Erdemir: Writing of the manuscript or critical review of important intellectual content; effective participation in the research guidance; intellectual participation in the propaedeutic and/or therapeutic conduct of the studied cases; final approval of the final version of the manuscript.

Ozan Erdem: Data collection, or analysis and interpretation of data; statistical analysis; writing of the manuscript or critical review of important intellectual content; critical review of the literature; final approval of the final version of the manuscript.

Elif Bal Avcı: Data collection, or analysis and interpretation of data; critical review of the literature; final approval of the final version of the manuscript.

Mehmet Salih Gürel: Study concept and design; effective participation in the research guidance; intellectual participation in the propaedeutic and/or therapeutic conduct of the studied cases; final approval of the final version of the manuscript.

## Conflicts of interest

None declared.
